# Volumetric assessment of individual thalamic nuclei in patients with drug-naïve, first-episode major depressive disorder

**DOI:** 10.3389/fpsyt.2023.1151551

**Published:** 2023-03-22

**Authors:** Enkhmurun Chibaatar, Keita Watanabe, Naomichi Okamoto, Nasanbadrakh Orkhonselenge, Tomoya Natsuyama, Gaku Hayakawa, Atsuko Ikenouchi, Shingo Kakeda, Reiji Yoshimura

**Affiliations:** ^1^Department of Psychiatry, University of Occupational and Environmental Health, Kitakyushu, Japan; ^2^Open Innovation Institute, Kyoto University, Kyoto, Japan; ^3^Department of Second Internal Medicine, University of Occupational and Environmental Health, Kitakyushu, Japan; ^4^Department of Radiology, Graduate School of Medicine, Hirosaki University, Hirosaki, Japan

**Keywords:** major depressive disorder, thalamus, thalamic nuclei volumes, subregional difference, structural MRI

## Abstract

**Introduction:**

Despite the previous inconsistent findings of structural and functional abnormalities of the thalamus in patients with major depressive disorder (MDD), the disruption of the thalamic nuclei in the pathophysiology of this disorder has not yet been adequately studied. Therefore, we investigated the volumetric changes of thalamic subregions and their nuclei in drug-naïve, first-episode MDD patients. We also investigated the association between HAM-D scores, a clinical scale frequently used to evaluate the severity of depression and thalamic nuclei volumes in MDD patients.

**Methods:**

This study included 76 drug-naïve MDD patients and an equal number of healthy subjects. Magnetic resonance imaging (MRI) data were obtained using a 3T MR system and thalamic nuclei volumes were evaluated using FreeSurfer ver.7.11. The volumetric differences were compared by one-way analysis of covariance (ANCOVA) and to ensure that effects were not accounted for by other factors, age, sex, and ETICV variables were included as covariates.

**Results:**

We observed significant volume reductions of the left whole thalamus (*p* < 0.003) and several thalamic nuclei mostly on the left side in the MDD group compared with healthy controls (HCs). Furthermore, we have revealed weak negative correlations between several thalamic nuclei volumes and HAM-D total and subscale scores.

**Discussion:**

This is the first research study to investigate alterations of the various thalamic nuclei volumes in MDD patients compared with HCs. Moreover, we first analyzed the association between individual thalamic nuclei volumes and HAM-D subscale scores. Though our study may be restricted at certain levels, especially by the demographic difference between the two groups, they possibly contribute at a preliminary level to understanding the thalamic structural changes at its subregions in patients with drug-naïve, first-episode MDD.

## 1. Introduction

Major depressive disorder (MDD) is a highly devitalizing mental disease that is common, with a high lifetime prevalence of approximately 15–20% (1). Patients with MDD are bothered by irritable and empty feelings, loss of pleasure or interest, impaired cognitive function, and, most importantly, thoughts of death or suicide (2). Specific structural alterations in brain regions and circuits, including the cortico-striato-thalamo-cortical (CSTC) circuit, have been reported as pathophysiological models of MDD that explain impairments in emotional and cognitive processes (3, 4). Among the brain regions that show deterioration and involvement in the circuit, the thalamus has been a prominent topic because of its extensive connections with other areas and its critical roles in cognitive impairment (5, 6). Although the thalamus is formed into functionally segregated nuclei, each with distinct anatomical locations and physiological functions (7–9), disruption of the thalamic nuclei in MDD patients has not yet been adequately studied.

Recent structural magnetic resonance imaging (MRI) studies have revealed inconsistent findings regarding the thalamic structure in patients with first-episode MDD. While most of the studies have shown a reduced volume in the bilateral thalami (10–14) or the left thalamus (15, 16), other meta-analyses observed increased thalamic volumes in patients with MDD (17, 18). As a volumetric change in the whole thalamus does not inform us about the functional significance of the cognitive and emotional processes underlying the pathophysiology of MDD, only one study has investigated thalamic subregions in the context of MDD. Choi et al. reported contractions in the medial and lateral nuclei in participants with MDD compared with participants in the control group (19). However, a more comprehensive segmentation study of the thalamus is required to identify the potential role of specific thalamic nuclei in the pathophysiology of MDD. Determining whether specific nuclei primarily drive thalamic differences could pinpoint specific circuits more affected in MDD, providing a potential avenue for targeted treatment strategies.

*In vivo* estimation of thalamic nuclei volumes could be challenging because of the limited contrast of most structural MRI images, which affects the accurate delineation of the internuclear borders. However, recent advances in automated thalamic segmentation have allowed unbiased large-scale volumetric analysis of individual nuclei. For instance, a probabilistic atlas presented by Iglesias et al. (20) enabled automatic segmentation of the thalamus into 25 specific nuclei using *ex vivo* brain MRI, histological data, and an *in vivo* MRI segmentation atlas.

In the present study, we applied this method to investigate volumetric differences in six different thalamic subregions (anterior, lateral, ventral, intralaminar, medial, and posterior) between drug-naïve patients with MDD in their first episode and healthy controls (HCs). We also investigated the association between HAMD-17 scores, a clinical scale frequently used to evaluate the severity of depression, and thalamic nuclei volumes in patients with MDD. As this is the first study of volumetric differences within various nuclei of the thalamus in the context of MDD and all nuclei can be potentially relevant from a functional perspective, we did not postulate an *a priori* hypothesis regarding which nuclei show differences.

## 2. Materials and methods

### 2.1. Participants

Seventy-six drug-naïve MDD patients (34 males, 42 females) and seventy-six healthy subjects (50 males, 26 females) participated in this study (Table 1). All patients with MDD were recruited from the inpatient and outpatient clinics of the Department of Psychiatry at the University of Occupational and Environmental Health (UOEH) Hospital, according to the following criteria: (1) newly diagnosed with MDD based on standard criteria of the Diagnostic and Statistical Manual of Mental Disorders–5 (DSM-V); (2) scored equal to or more than 14 on the 17-items Hamilton Rating Scale for Depression (HAMD-17); (3) did not use antidepressants or other psychiatric drugs; and (4) had no previous history of medical illness, neurological, or psychiatric disorders. Healthy subjects were recruited from the community through advertisements according to the following criteria: (1) had never been diagnosed with any psychiatric disorders according to the Structured Clinical Interview for DSM Disorders (SCID), and (2) had no family history of a serious medical or neuropsychiatric disorder among their first-degree relatives. All participants provided written informed consent after the study procedure was explained. The study protocol was approved by the Ethics Committee of the UOEH and was conducted in accordance with the Declaration of Helsinki.

**TABLE 1:** Demographic and clinical characteristics of patients with MDD and healthy controls.

Characteristics	Patients with MDD (*n* = 76)	Healthy controls (*n* = 76)
Age	53.71 17.07	35.66 ± 12.07
**Gender**		
Male (*n*, %)	34 (44.74%)	50 (65.79%)
Female (*n*, %)	42 (55.26%)	26 (34.21%)
ETICV (mm^3^ ± SD)	15.21 10^5^ ± 1.74 10^5^	15.89 10^5^ ± 1.36 10^5^
HAMD-17 total score	22.45 6.283	–
**HAMD-17 subscale score**		
Core (0–22)	10.18 ± 3.56	–
Sleep (0–4)	2.43 ± 1.13	–
Activity (0–8)	3.82 ± 1.51	–
Psychic (0–8)	2.78 ± 1.28	–
Anxiety (0–6)	2.71 ± 1.20	–

Values represent the mean ± SD unless otherwise stated. MDD, major depressive disorder; ETICV, estimated total intracranial cavity volume; HAMD-17, 17 items hamilton depression rating scale.

### 2.2. Hamilton rating scale for depression

Hamilton Rating Scale for Depression HAMD-17 items were divided into the following subcategories: core (items 1, 2, 7, 8, 10, and 13), sleep (items 4 and 6), activity (items 7 and 8), psychic (items 9 and 10), and somatic anxiety (items 11 and 13), similar to our previous work (21, 22).

### 2.3. Structural MRI acquisition

Magnetic resonance imaging data were obtained using a 3T MR system (Signa EXCITE 3T; GE Healthcare, Waukesha, WI, USA) with an 8-channel brain phased-array coil. Images were acquired using three-dimensional fast-spoiled gradient-recalled acquisition (3D-FSPGR). The acquisition parameters were as follows: repetition time/echo time, 10/4.1 msec; flip angle, 10°; field of view, 24 cm; resolution, 0.9 mm × 0.9 mm × 1.2 mm. All images were corrected for image distortion due to gradient non-linearity using the “Grad Warp” software program (23).

### 2.4. Image processing (thalamic segmentation)

**FIGURE 1:**
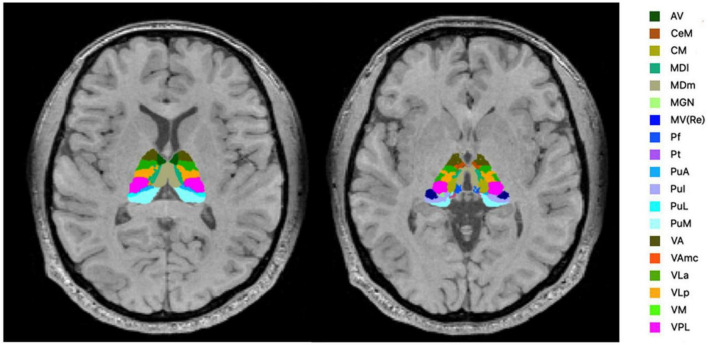
Segmentation of the thalamic nuclei. The example of probabilistic segmentation (not all segmentations are shown) of the thalamus generated by FreeSurfer ver.7.11 (23). Anterior region: AV, anteroventral. Intralaminar region: CeM, central medial; CM, centromedian; Pf, Parafascicular; medial region: MDI, mediodorsal lateral parvocellular; MDm, mediodorsal medial magnocellular; MV-re, medial ventral reuniens; Pt, paratenial. Posterior region: MGN, medial geniculate; PuA, pulvinar anterior; PuI, pulvinar inferior; PuL, pulvinar lateral; PuM, pulvinar medial. Ventral region: VA, ventral anterior; VAmc, ventral anterior magnocellular; VLa, ventral lateral anterior; VLp, ventral lateral posterior; VM, ventromedial; VPL, ventral posterolateral.

FreeSurfer ver.7.11 (24) was used to evaluate the volume of thalamic subregions. This fully automated segmentation technique for thalamic nuclei is based on a prior probabilistic atlas and a Bayesian modeling approach (20). The bilateral thalami were generated in each subject for 25 nuclei in six different regions (Figure 1) and the whole thalamus. The left and right substructures were analyzed separately. Furthermore, the estimated intracranial volume was also calculated using “Aseg segmentation.”

### 2.5. Statistical analyses

All statistical analyses were performed using IBM SPSS Statistics (version 26.0; SPSS Inc., Chicago, IL, USA). We performed independent *t*-tests and *x*^2^ tests to compare the demographic and clinical data between patients with MDD and HCs. To investigate the volumetric differences, a one-way analysis of covariance (ANCOVA) was used to ensure that the effects were not accounted for by other factors; age, sex, and ETICV variables were included as covariates. The assumptions of normal distribution, linearity, and homogeneity of variance were tested and verified. The Bonferroni’s correction method was used for multiple comparisons. To examine the association between thalamic nuclei volumes and HAMD-17 scores, we performed a partial correlation analysis with age, sex, and ETICV as covariates. Results were considered statistically significant at *p* < 0.05.

## 3. Results

### 3.1. Demographic and clinical data

The demographic and clinical characteristics of patients with MDD and HCs are shown in Table 1. Significant differences (*p* < 0.05) were observed between the groups in age, sex, and estimated total intracranial volume (ETICV). However, these variables were used as covariates to eliminate their effects in further analyses. Table 1 also showed HAM-D total and subscale scores in the MDD group. Notably, all patients with MDD were in the first episode and were medication-free.

### 3.2. Volumetric analysis

**FIGURE 2:**
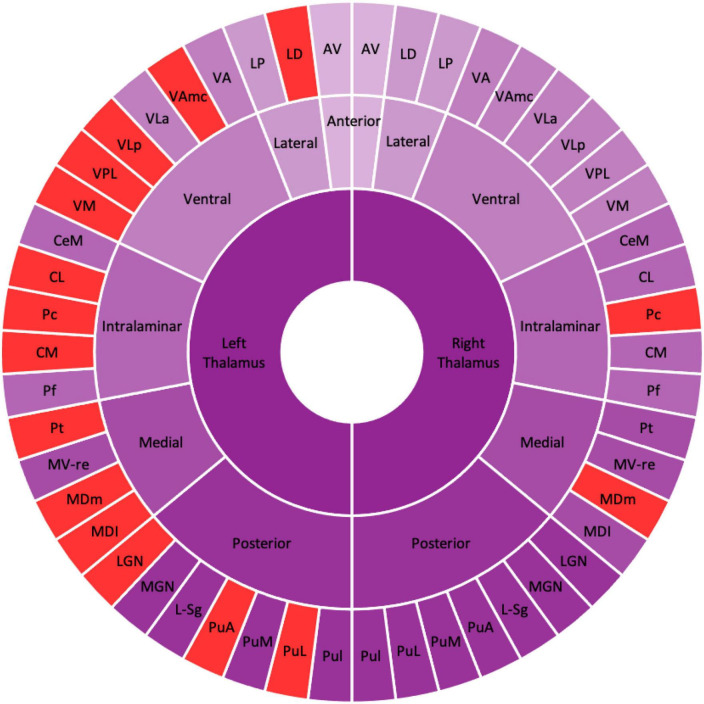
Schematic figure of the altered thalamic nuclei. Schematic illustration of altered thalamic nuclei. The nuclei in the red represent the significant volume alteration in patients with MDD as compared with HCs. The figure shows the left thalamic nuclei are significantly reduced compared with the right thalamic nuclei. AV, anteroventral; LD, laterodorsal; LP, lateral posterior; VA, ventral anterior; VAmc, ventral anterior magnocellular; VLa, ventral lateral anterior; VLp, ventral lateral posterior; VPL, ventral posterolateral; VM, ventromedial; CeM, central medial, CL, central lateral; Pc, paracentral; CM, centromedian; Pf, parafascicular; Pt, paratenial; MV-re, medial ventral reuniens; MDm, mediodorsal medial magnocellular; MDI, mediodorsal lateral parvocellular; LGN, lateral geniculate; MGN, medial geniculate; L-Sg, limitans suprageniculate; PuA, pulvinar anterior; PuM, pulvinar medial; PuL, pulvinar lateral; PuI, pulvinar inferior.

**FIGURE 3:**
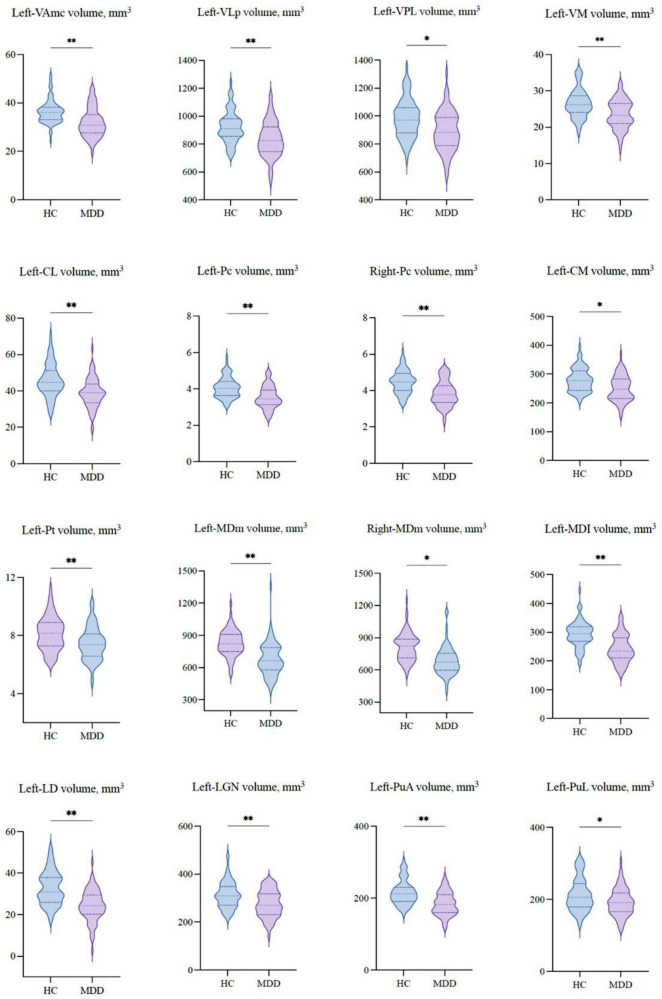
The volumetric differences of thalamic nuclei between MDD patients and HCs. Significant volume differences between groups. *p*-values were obtained using a one-way analysis of covariance (ANCOVA) adjusted for age, sex, and estimated total intracranial cavity volume (ETICV) as covariates. The Bonferroni’s correction was applied, *p < 0.05 and **p < 0.03.

Table 2 shows the thalamic volume as a whole and the individual nuclei. The right thalamic volume was not different between the two groups of patients with MDD and HCs; in contrast, the left thalamus was significantly different (*p* < 0.003). Regarding individual nuclei, significant differences were detected in 16 nuclei belonging to five regions, including the lateral, ventral, intralaminar, medial, and posterior regions, in which we found volume reductions in patients with MDD. Intergroup volumetric changes showing significance are summarized in Figures 2, 3. We found a significant bilateral volumetric decrease in the Pc nuclei of the intralaminar regions (L: *p* = 0.01; R: *p* = 0.03) and MDm nuclei of the medial regions (L: *p* = 0.01; R: *p* = 0.05). We also observed significant volumetric reductions in several nuclei of the left thalamus. Specifically, Left-LD (*p* < 0.001) in the lateral region, left VAmc (*p* = 0.03), left VLp (*p* = 0.03), left VPL (*p* = 0.04), and left VM (*p* = 0.02) in the ventral region, left CL (*p* = 0.03), left CM (*p* = 0.04) in the intralaminar region, left Pt (*p* = 0.03), and left MDI (*p* = 0.01) in the medial region. Last of all, left-LGN (*p* = 0.03), left-PuA (*p* = 0.02), and left-PuL (*p* = 0.04) in the posterior region.

**TABLE 2:** Differences in thalamic nuclei volumes between patients with MDD and healthy control.

Thalamic nucleus	Patients with MDD (*n* = 76)		Healthy controls (*n* = 76)		*F*	*p*-value
	Mean, mm^3^	SD, mm^3^	Mean, mm^3^	SD, mm^3^		
Left whole thalamus	6425.39	865.44	7293.72	795.60	8.91	0.003*
Right whole thalamus	6493.89	805.46	7170.51	747.52	2.88	0.092
**Anterior**						
Left-AV	137.96	23.19	151.09	24.50	2.73	0.101
Right-AV	145.07	26.32	158.22	25.36	3.10	0.080
**Lateral**						
Left-LD	24.31	7.47	32.71	8.04	13.84	<0.001*
Right-LD	28.44	8.57	33.91	8.60	1.69	0.195
Left-LP	124.01	24.66	141.73	19.73	3.09	0.081
Right-LP	117.41	24.75	134.81	20.77	2.63	0.107
**Ventral**						
Left-VA	404.61	68.30	449.40	61.21	2.44	0.121
Right-VA	377.08	60.65	421.18	54.23	1.97	0.162
Left-VAmc	31.89	5.93	36.36	4.83	5.05	0.026*
Right-VAmc	32.16	5.73	36.01	4.67	1.40	0.238
Left-VLa	636.55	103.85	701.07	90.93	3.37	0.069
Right-VLa	612.95	96.15	672.73	88.98	1.34	0.248
Left-VLp	835.66	126.17	922.67	112.70	4.66	0.033*
Right-VLp	799.73	118.25	882.43	111.00	2.04	0.155
Left-VPL	892.78	142.20	984.15	137.13	4.51	0.035*
Right-VPL	844.68	127.75	935.08	127.83	2.55	0.112
Left-VM	23.57	3.93	26.51	3.93	5.49	0.020*
Right-VM	22.71	3.71	25.01	3.55	1.19	0.278
**Intralaminar**						
Left-CeM	66.41	13.52	73.25	12.26	1.34	0.249
Right-CeM	70.38	14.87	75.53	11.52	0.32	0.572
Left-CL	38.84	8.04	45.76	9.20	4.84	0.029*
Right-CL	40.96	9.48	46.35	10.92	0.85	0.359
Left-Pc	3.54	0.61	4.07	0.57	6.20	0.014*
Right-Pc	3.84	0.69	4.47	0.63	4.69	0.032*
Left-CM	250.08	44.34	279.85	40.62	4.31	0.040*
Right-CM	241.02	39.88	264.69	41.00	1.19	0.276
Left-Pf	59.70	11.09	67.40	10.21	3.18	0.077
Right-Pf	61.66	12.35	67.57	13.85	0.95	0.332
**Medial**						
Left-Pt	7.44	1.13	8.13	1.16	4.88	0.029*
Right-Pt	7.35	1.13	8.08	1.05	2.34	0.128
Left-MV-re	11.64	3.30	13.77	3.24	1.28	0.260
Right-Mv-re	11.92	3.71	13.79	3.40	0.14	0.706
Left-MDm	678.72	153.81	828.76	119.61	6.39	0.013*
Right-MDm	683.59	130.05	814.32	129.87	4.09	0.045*
Left-MDI	242.05	48.06	292.95	45.27	6.48	0.012*
Right-MDI	247.92	50.93	291.45	52.47	1.81	0.181
**Posterior**						
Left-LGN	275.52	55.69	313.47	55.25	5.09	0.026*
Right-LGN	267.60	59.66	304.56	53.34	2.29	0.132
Left-MGN	118.09	22.45	126.93	21.94	0.00	0.989
Right-MGN	120.01	25.67	128.33	23.17	0.04	0.841
Left-L-Sg	24.70	7.74	28.56	7.36	0.10	0.749
Right-L-Sg	19.76	7.44	23.17	8.03	1.24	0.267
Left-PuA	184.25	32.09	215.47	34.22	5.57	0.020*
Right-PuA	200.68	29.13	218.36	24.58	0.11	0.739
Left-PuM	939.24	169.40	1087.96	195.19	3.52	0.062
Right-PuM	1062.58	156.02	1135.65	146.29	0.00	0.947
Left-PuL	192.74	38.20	213.04	45.61	4.28	0.040*
Right-PuL	219.68	46.29	215.71	49.36	0.13	0.717
Left-PuI	221.10	46.93	248.66	53.71	2.20	0.140
Right-PuI	254.70	49.88	259.09	53.25	0.03	0.873

AV, anteroventral; LD, laterodorsal; LP, lateral posterior; VA, ventral anterior; VAmc, ventral anterior magnocellular; VLa, ventral lateral anterior; VLp, ventral lateral posterior; VPL, ventral posterolateral; VM, ventromedial; CeM, central medial, CL, central lateral; Pc, paracentral; CM, centromedian; Pf, parafascicular; Pt, paratenial; MV-re, medial ventral reuniens; MDm, mediodorsal medial magnocellular; MDI, mediodorsal lateral parvocellular; LGN, lateral geniculate; MGN, medial geniculate; L-Sg, limitans suprageniculate; PuA, pulvinar anterior; PuM, pulvinar medial; PuL, pulvinar lateral; PuI, pulvinar Inferior. The F and p-values were obtained using a one-way analysis of covariance (ANCOVA) adjusted for age, sex, and estimated total intracranial cavity volume (ETICV) as covariates. The Bonferroni’s correction was applied, *p < 0.05.

### 3.3. Correlation analysis

**FIGURE 4:**
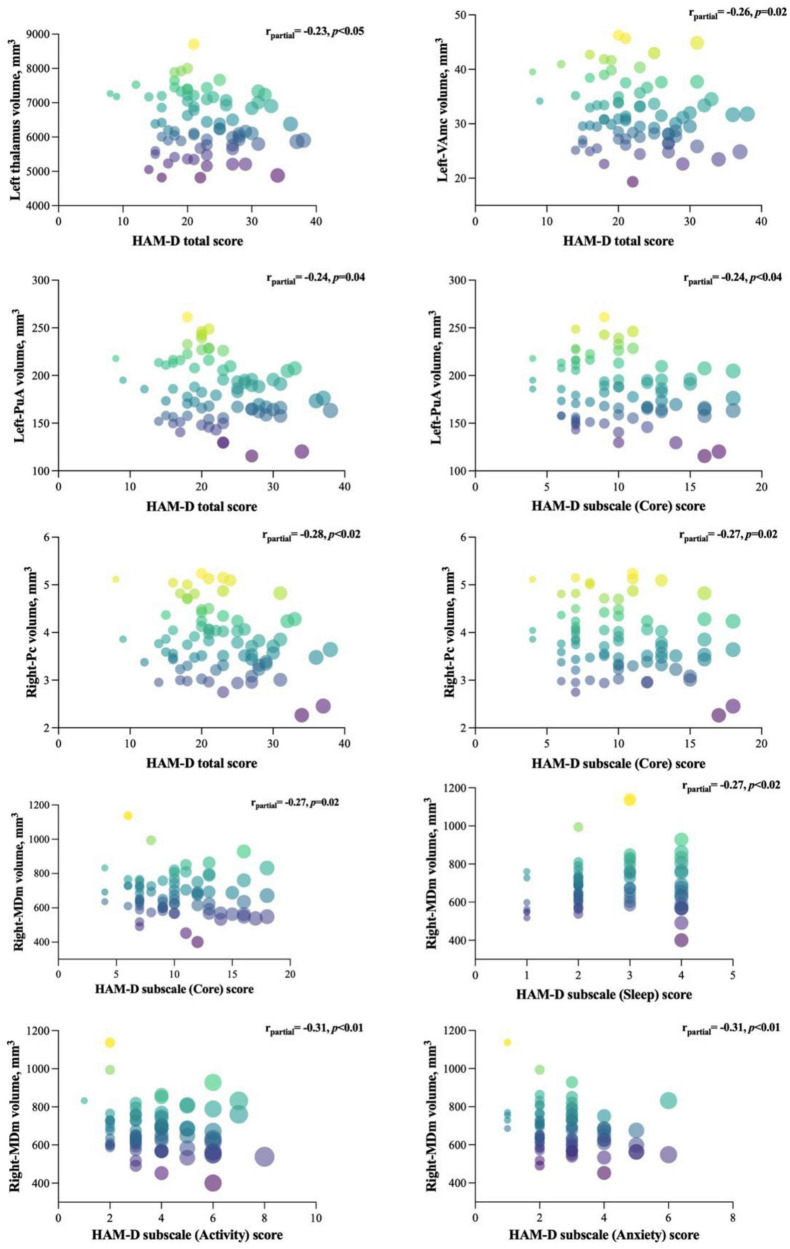
Significant correlations between the affected nuclei volume and HAMD-17 total and subscale scores. The figure represents the correlation between affected nuclei volume and depression severity, as assessed by the HAMD-17 score.

The results of partial correlation analyses between affected nuclei volume and depression severity, as assessed by the HAMD-17 score, are shown in Figure 4. We found that the HAMD-17 total score was negatively correlated with the volume of the left thalamus (*r* = −0.232, *p* = 0.05), VAmc (L: *r* = −0.262, *p* = 0.03), and PuA (*r* = –0.241, *p* = 0.04) in the left thalamus, and Pc (*r* = −0.293, *p* = 0.01) in the right thalamus. Moreover, we assessed the relationship between affected nuclei and HAMD-17 subscale scores. Only three nuclei showed a negative correlation. The right Pc was correlated with the core score (*r* = −0.267, *p* = 0.02), and the left-PuA was associated with the core (*r* = −0.243, *p* = 0.04) and activity scores (*r* = −0.247, *p* = 0.04). Interestingly, only the right-MDm was negatively correlated with core (*r* = −0.270, *p* = 0.02), sleep (*r* = −0.274, *p* = 0.02), activity (*r* = −0.309, *p* = 0.01), and anxiety (*r* = −0.306, *p* = 0.01) scores.

## 4. Discussion

In the present study, volumetric changes in various nuclei in six different regions of the bilateral thalami were investigated in medication-free, first-episode MDD patients relative to HCs. We observed significant volume reductions in the left thalamus and several thalamic nuclei, mainly on the left side, in the MDD group. Furthermore, we revealed weak negative correlations between several thalamic nuclei volumes and the HAMD-17 total and subscale scores.

As emerging perspectives on the thalamus and its role in the neuropathology of depression, several studies have investigated thalamic volume in patients with MDD. Following the earliest study that reported significant bilateral thalamic volume reduction in female subjects diagnosed with MDD (10), assorted studies have confirmed this result in different subjects with MDD (11–14, 19), including older adults (10, 14), patients with mild symptoms (13), and drug naïve MDD patients (19). In this study, we found thalamic volume contraction only on the left side, which is consistent with some volumetric studies in recent years (15, 16). However, these studies have several limitations compared to our research. They analyzed the thalamus as a whole or segmented it into subregions while we investigated the alterations of 25 individual thalamic nuclei volumes. Even so, previous meta-analyses that enrolled drug-naïve MDD patients revealed significantly increased gray matter volume in the thalamus (17, 18), contradictory to our findings. All participants with MDD in our study were medication-free, and in their first episode, like those in the meta-analyses, their average age was higher (53.7) compared with MDD patients in the previous studies. Therefore, these opposing results of increased volumetric changes in the thalamus may be specifically related to the patient’s age and could reflect early thalamic hyperfunction (14).

According to thalamic nuclei functions, some nuclear groups are known as relay stations. They receive specific and well-defined motor and sensory information inputs and project them to the brain cortex. Previous functional studies have reported that the lateral and ventral nuclei of the thalamus process motor and somatosensory information and support alertness and arousal in humans (25–27), as well as in rodents (28). The importance of the posterior region, especially the LGN, was also reported in a recent meta-analysis (29). In the present study, we found significant reductions in LD nuclei in the lateral region, VAmc, VLp, VPL, and VM nuclei in the ventral region, and LGN in the posterior region of the left thalamus in patients with MDD compared to HCs. Given that physical pain and psychomotor retardation or agitation often present symptoms of MDD (30, 31), we assumed that contractions in these nuclei could be related to the above-mentioned somatic symptoms. However, further functional and connectivity studies are required to provide further insight.

Intralaminar nuclei, known as “non-specific” nuclei of the thalamus, seem to be broadly connected with the entire cortex and globally activate it (32). In the current study, we found that patients with MDD had significantly decreased intralaminar nuclei of the CL and CM in the left thalamus and Pc in the bilateral thalamus. However, it is not clear whether volume reductions in these nuclei play a crucial role in MDD, and the concept that intralaminar nuclei can facilitate some depression symptoms is consistent with the findings of previous studies indicating that lesions to the intralaminar nuclei lead to attention diminishment. For example, Van Der Werf et al. (33) reported that damage to this area elicits complex attention deficits. Furthermore, other findings detailed the contribution of rostral intralaminar nuclei, together with CL, Pc, and CM, to extended cognitive and behavioral functions (34–36). Relating to the major depression symptoms of insufficient concentration and indecisiveness, our results of depletion in rostral intralaminar nuclei, specifically Pc nuclei in the bilateral thalamus in MDD patients, consistently support prior outcomes, such as the influence of cognitive processes (37), impairment of working memory (38), and attentional engagement of sensory events (35). Thus, our findings suggest that alterations in these rostral intralaminar nuclei could be related to depression-related arousal, awareness, and attention deficits in patients with MDD.

Among the thalamus parts that showed differences in patients with MDD, the medial and pulvinar have been the regions of interest because of their significant interconnection with the prefrontal cortex and other subcortical structures. The results of the study of many neuropsychiatric disorders reported changes in these regions of the thalamus, particularly in obsessive-compulsive disorder (OCD) (39, 40), psychosis (41), schizophrenia (42, 43), and Parkinson’s disease (44). As for depression, few studies have investigated the structural (19) and functional (45) alterations of medial and pulvinar regions in MDD patients. Although the results of these previous studies were consistent with the outcomes of our research, none of them investigated detailed structural changes in the different nuclei of the thalamus. In particular, the mediodorsal (MD) nuclei and pulvinar nuclei (Pu). MD, one of the largest nuclei of the thalamus, is primarily involved in emotional, cognitive, and behavioral processing and is often impaired in depression (1). We found altered MDm, Pt, and MDI nucleus volumes in the current study. Above all, only the MDm nucleus showed differences on both sides of the thalamus, whereas the remaining two were on the left side. Given that the MD nuclei or its medial part physiologically interact with the prefrontal cortex during cognitive function (46) and emotion regulation (24), our findings suggest that alterations in the MD nuclei could be directly related to emotional and behavioral failure in MDD through involvement in the orbitofrontal circuit. Indeed, we observed volume reductions in the PuA and PuL nuclei of the left thalamus. Pu, the largest nuclear mass, is involved in executive function and emotional processing (47). Moreover, its circuit with the cortex predicts cognition in many psychiatric disorders, including schizophrenia (48) and psychosis (41).

Similarly, it showed a positive response to antidepressant treatment (49, 50). The Pu nuclei also play a role in attention (51), indicating that it could influence the dysfunctional cognition process and abnormal emotional behaviors accompanying MDD; whether the relationship between depression and structural changes in the thalamic nuclei in MDD remains a critical argument that requires further specifically designed approaches.

We also investigated the correlations between the HAMD-17 total and subscale scores and the affected thalamic nuclei volumes in patients with MDD. We found a weak negative correlation between the left thalamus and HAMD-17 total score, which supports previous reports of an association between thalamic volume and depression severity (52). Moreover, in the left thalamus, VAmc and PuA nuclei were negatively associated with evaluation scores. These findings suggest that the nuclei of the ventral and posterior regions of the thalamus are negatively correlated with depression severity, suggesting their involvement in the anterior cingulate-prefrontal circuit (53). Regarding HAMD-17 subscale scores, the PuA nucleus of the left thalamus and Pc and MDm nuclei of the right thalamus showed a weak negative correlation with the core score, which might suggest their role in the core symptoms of depression, including depressed mood, loss of energy, and difficulty with memory and attention (45). Interestingly, only the right MDm nucleus was negatively correlated with sleep, activity, and anxiety scores. As the MDm nuclei have extensive efferent and afferent connections with the prefrontal cortex, motor cortex, basal ganglia, and amygdala, this result suggests that structural changes in the MDm nuclei may be relevant for explaining MDD symptoms, which are poorly understood (54). In support of this proposal, future studies will benefit from using longitudinal approaches that investigate the functional and structural relationships between specific thalamic nuclei and the clinical symptoms of MDD.

To the best of our knowledge, this is the first MDD study to investigate alterations in the volumes of various thalamic nuclei compared with HCs. Moreover, we first analyzed the association between individual thalamic nuclei volumes and HAMD-17 subscale scores. Nevertheless, this study had several limitations that should be addressed. First, we used a cross-sectional design, which makes it challenging to investigate the causal association between structural changes and clinical features. Second, we enrolled a relatively small sample without age and sex matching, limiting the generalizability of our results. Even though the analysis was statistically adjusted, demographic indicators between the two groups were different. Therefore, to understand the role of different regions of the thalamus in the pathophysiology of depression, longitudinal studies with larger age- and gender-matched sample sizes are required. Third, we did not investigate detailed clinical data, including handedness, educational year, age of onset, and duration of illness, which might have influenced the volumetric changes we reported. Finally, the present volumetric study did not point to functional abnormalities or neuronal plasticity associated with depression; thus, additional work is required to elucidate alternative explanations for the connections between structural and functional pathology and depressive symptoms.

## 5. Conclusion

In summary, our findings showed a reduction in the volume of the left thalamus in medication-free patients with MDD. The volumetric data of specific thalamic nuclei also showed alterations, mainly on the left side. In addition, our results also highlight the relationship between thalamic nuclei volume and the severity of clinical symptoms, suggesting an association between volumetric alteration of various nuclei in the thalamus relevant to the clinical outcome of depression. Although these findings may be restricted to certain levels, they may contribute to understanding brain structural changes in MDD and highlight the need for further investigation.

## Data availability statement

The raw data supporting the conclusions of this article will be made available by the authors, without undue reservation.

## Ethics statement

The studies involving human participants were reviewed and approved by the Ethics Committee of the University of Occupational and Environmental Health, Japan. The patients/participants provided their written informed consent to participate in this study.

## Author contributions

EC, KW, SK, and RY: study conception and design. KW, NOk, TN, GH, AI, SK, and RY: data collection. EC, KW, and NOr: analysis and interpretation of results. EC: draft manuscript preparation. All authors reviewed the results and approved the final version of the manuscript.
